# The complete mitogenome of the *Paranticopsis xenocles* (Lepidoptera: Papilionidae: Papilioninae) and phylogenetic implications

**DOI:** 10.1080/23802359.2021.1997113

**Published:** 2021-11-03

**Authors:** Zhenhuai Fan, Yaping Hu, Site Luo, Danlan Hu, Xueqian Wang, Wenbo Fu, Bin Chen, Zhentian Yan

**Affiliations:** a Chongqing Key Laboratory of Vector Insects; Institute of Entomology and Molecular Biology, Chongqing Normal University, Chongqing, China; b Ministry of Ecology and Environment, Nanjing Institute of Environmental Sciences, Nanjing, China; c School of Life Sciences, Xiamen University, Xiamen University, Xiamen, China

**Keywords:** *Paranticopsis xenocles*, papilionidae, mitochondrial genome, phylogenetic relationship

## Abstract

*Paranticopsis xenocles* Doubleday belongs to the *Paranticopsis* of Papilionidae and is mainly distributed in China mainland. Herein, we report the complete mitogenome of *P. xenocles* reconstructing from Illumina sequence data. The mitogenome is 15,187 bp in length and contains 13 protein-coding genes, 22 transfer RNA genes, and 2 ribosomal RNA genes. The phylogenetic analysis indicated that *P. xenocles* were clustered within *Paranticopsis*. This study would provide useful genetic information for future studies on taxonomy, phylogeny, and evolution of Papilionidae species.

*Paranticopsis xenocles* Doubleday (Doubleday 1842) belongs to the *Paranticopsis* of Papilionidae. It was mainly distributed in the Hainan and Yunnan provinces of China, occasionally seen in Guangxi and Guangdong. It is also found in India, Bhutan, Myanmar, Thailand, and Vietnam (Wu 2001). In previous butterfly research, there were no morphological or molecular studies on this butterfly. There are 20 species of the genus *Paranticopsis* recorded in the world, most of which are distributed in the Oriental region, and four species distributes in China (Wu 2001). At present, molecular markers (*COI*, *COII*, *ND5*, *Cytb*, and *16S rDNA*) are widely used in the molecular phylogenetic studies of butterflies (Qin 2017). The *16S rDNA* and *Cytb* genes are relatively conserved (Torres et al. 2001), while the evolution rate of *COI*, *COII*, *ND1,* and *ND5* genes is relatively fast (Brunton and Hursth 1998; Reed and Sperling 1999). Due to the different mutation rates of different mtDNA gene sequences, different genes are suitable for analyzing the phylogenetic relationships of different taxonomic levels (Yuan and Yuan 2013). Currently, no study had been published on the complete mitogenome sequence of *P. xenocles*. Here we performed high-throughput sequencing on a specimen of *P. xenocles* from China to determine its mitogenome structure and evolutionary relationship between it and other 10 Papilionidae species.

The species sample was collected at the Guaifengkou of Chongqing Simian Mountain Nature Reserve in Jiangjin, China (28°46′51″N, 106°19′23″E). The voucher specimen is deposited at Chongqing Normal University (No. 20190816006, Zhentian YAN: 525201877@qq.com). The genomic DNA was extracted by using TIANamp Genomic DNA Kit (TIANGEN, Beijing, China). The sequencing library was produced by using the Illumina Truseq™ DNA Sample Preparation Kit (Illumina, San Diego, USA) according to the manufacturer's recommendations. The prepared library was loaded on the Illumina Novaseq 6000 platform for PE 2 × 150 bp sequencing at Novogene (Beijing, China). The raw data were used to assemble the complete mitochondrial genome using the GetOrganelle pipeline (Jin et al. 2020). Genome annotation was performed with the Mitoz annotation module (Meng et al. 2019). The annotated genome sequence was deposited in GenBank under Accession Number MZ394042.

The complete mtgenomes of *P. xenocles* was 15,187 bp (GenBank number MZ394042) in length. It has thirty-seven typical mtgenome genes (13 protein-coding genes, 22 transfer RNAs, and 2 ribosomal RNAs genes). The mtgenomes of *P. xenocles* showed a High nucleotide bias with 80.17% of A + T and 19.83% of G + C (41.37% A;38.80% T;12.10% G; and 7.73% C).

Each of mitochondrial genes was separately aligned and concatenated by the MAFFT v7.388 with default settings (Katoh and Standley 2013). We constructed the phylogenetic relationship of mtgenomes of *P. xenocles* and 10 other Papilionidae species using the maximum likelihood criterion (ML) method with IQ-TREE v2.1.2 and with the *Polyura nepenthes* mtgenome (NC_026073) as an outgroup. The nucleotide sequences of the 13 PCGs were used in the phylogenetic analysis and the best model GTR + F+R2 was selected using ModelFinder for the analysis (Minh et al. 2020, Kalyaanamoorthy et al. 2017).

The support for the inferred ML tree was inferred by bootstrapping with 1,000 replicates. The analysis showed that *P. xenocles* was placed in a clade including other Papilioninae species (Figure 1). This study provides important sequence information for species identification and its phylogenetic position in Papilionidae.

**Figure 1.**
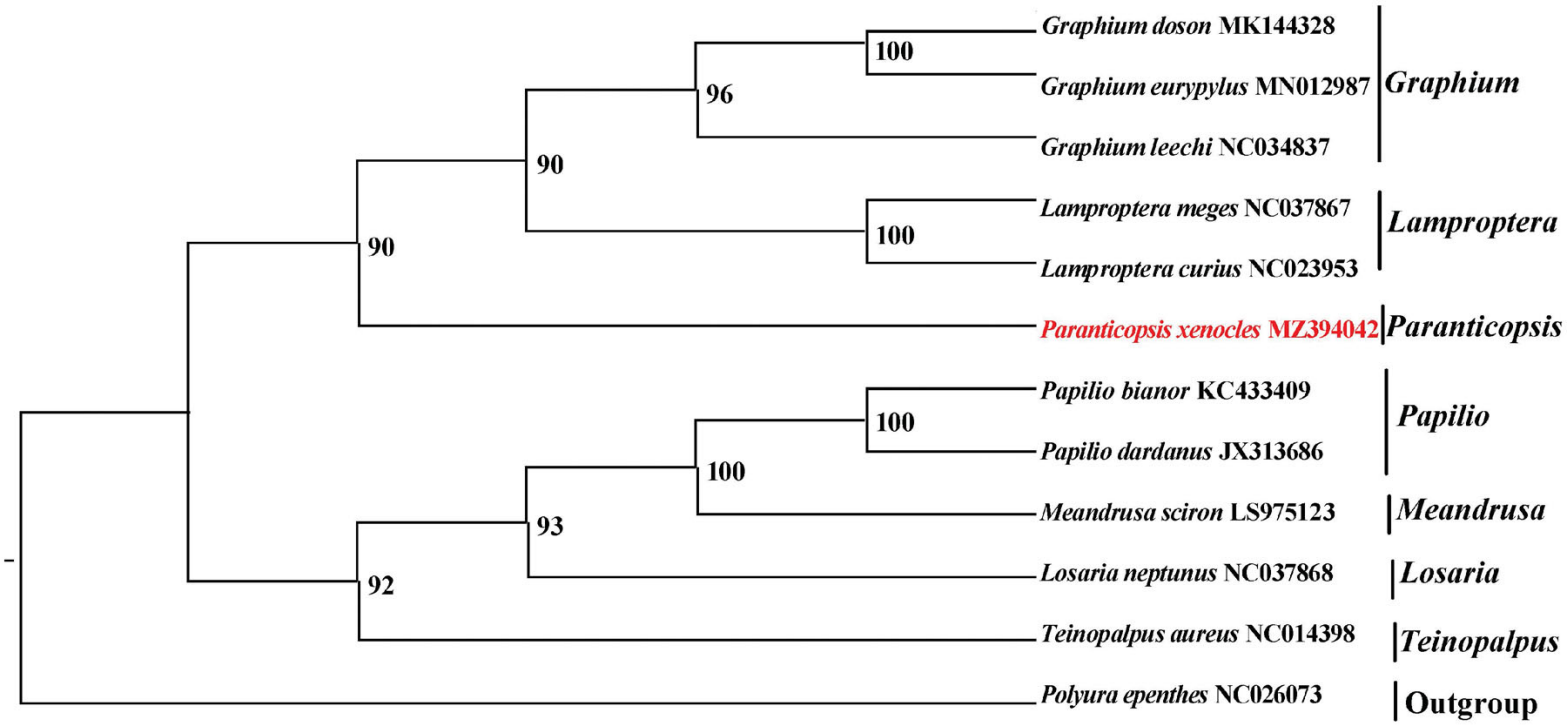
Maximum-likelihood (ML) tree based on 12 mitogenome sequences of representative butterflies that are in Papilioninae as ingroup and *Polyura nepenthes* was designated as the outgroup. Numbers on the nodes are bootstrap values based on 1,000 replicates. The *P. xenocles* genome was marked in bold and red font.

## Data Availability

The genome sequence data that support the findings of this study are openly available in GenBank of NCBI (https://www.ncbi.nlm.nih.gov/) under the accession no MZ394042. The associated BioProject, SRA, and Bio-Sample numbers are PRJNA724942, SAMN18865638, and SRR14325656, respectively.
